# Radiological findings associated with the death of newborns with necrotizing enterocolitis

**DOI:** 10.1590/0100-3984.2017.0040

**Published:** 2018

**Authors:** Isabela Gusson Galdino dos Santos, Maria Aparecida Mezzacappa, Beatriz Regina Alvares

**Affiliations:** 1 MD, Resident at the Faculdade de Ciências Médicas da Universidade Estadual de Campinas (FCM-Unicamp), Campinas, SP, Brazil.; 2 PhD, Professor in the Department of Pediatrics at the Faculdade de Ciências Médicas da Universidade Estadual de Campinas (FCM-Unicamp), Campinas, SP, Brazil.; 3 PhD, Professor in the Department of Radiology at the Faculdade de Ciências Médicas da Universidade Estadual de Campinas (FCM-Unicamp), Campinas, SP, Brazil.

**Keywords:** Enterocolitis, necrotizing/diagnosis, Infant, premature, Premature birth, Perinatal death

## Abstract

**Objective:**

The aim of this study was to identify radiological and clinical risk factors for death in newborns with necrotizing enterocolitis.

**Materials and Methods:**

This was a retrospective cohort study, based on radiological examinations and medical charts of 66 infants with necrotizing enterocolitis, as confirmed by a finding of intestinal pneumatosis (stage IIA, according to modified Bell’s staging criteria). Radiological and clinical variables were evaluated.

**Results:**

Of the 66 infants evaluated, 14 (21.2%) presented pneumatosis in the large and small bowel; 7 (10.6%) presented air in the portal system; and 12 (18.2%) died. Bivariate analysis revealed that the following variables were associated with death: bowel perforation; pneumatosis in the large and small bowel; air in the portal system; earlier gestational age; longer time on mechanical ventilation before the identification of pneumatosis; and longer time on mechanical ventilation before discharge or death. In the multivariate regression, the following variables remained as predictors of death: pneumatosis in the large and small intestines (odds ratio [OR] = 12.4; 95% confidence interval [95% CI] = 1.2-127.4; *p* = 0.035), perforation (OR = 23.2; 95% CI = 2.2-246.7; *p* = 0.009), and air in the portal system (OR = 69.7; 95% CI = 4.3-[not calculated]; *p* = 0.003).

**Conclusion:**

The set of factors most strongly associated with death in infants with necrotizing enterocolitis comprised extensive pneumatosis, pneumoperitoneum, and air in the portal system. Our findings confirm the importance of radiological imaging in the diagnosis and monitoring of necrotizing enterocolitis.

## INTRODUCTION

Necrotizing enterocolitis (NEC) is a severe inflammatory disease of the gastrointestinal tract that primarily affects newborns, accounting for 1-5% of all admissions to the neonatal intensive care unit^(^^1^^-^^3^^)^. Prematurity is the main risk factor, and more than 90% of affected newborns have a gestational age of less than 37 weeks^(^^1^^,^^2^^)^. This disease represents one of the main causes of surgical intervention among newborns, being associated with mortality rates of up to 50%^(^^2^^)^.

Conventional X-ray of the abdomen is the most commonly used imaging method for the diagnosis and follow-up of newborns with suspected or confirmed NEC, because of its ease of access and because it is a noninvasive procedure^(^^4^^)^. The earliest, albeit nonspecific, radiological sign is diffuse and asymmetrical distension of intestinal loops. The most specific findings are the presence of air in the portal venous system and intestinal pneumatosis, the latter being virtually pathognomonic for NEC^(^^4^^)^. More recently, abdominal ultrasound has been used for the diagnosis of NEC, because its sensitivity is greater than is that of conventional X-ray in some aspects, such as in the identification of free fluid in the abdominal cavity and in the determination of the thickness and the perfusion of the intestinal loops. However, ultrasound still has limited value in the prognostic evaluation of the disease and should be used as a complement to the X-ray examination^(^^4^^,^^5^^)^.

The prognostic value of radiological findings for the indication of surgery has been recognized^(^^6^^,^^7^^)^; however, their prognostic value for mortality has been little studied and is not consistent across studies^(^^8^^,^^9^^)^. 

The present study endeavors to evaluate clinical and radiological variables that behave as risk factors for the evolution to death in newborns with NEC, in order to contribute to the protocols of care for these patients.

## MATERIALS AND METHODS

This was a retrospective cohort study. The cases were selected based upon the X-ray examinations of newborns with NEC, diagnosed on the basis of clinical and radiological findings (intestinal pneumatosis), classified as stage IIA according to the modified Bell’s staging criteria^(^^10^^)^. The newborns had all been admitted to the neonatal intensive care unit of a teaching hospital between 1991 and 2013. Newborns referred from other facilities were excluded, as were those with congenital malformations. There were 66 cases that met the inclusion criteria. The study was approved by the local research ethics committee.

### Variables

**Figure 1:**
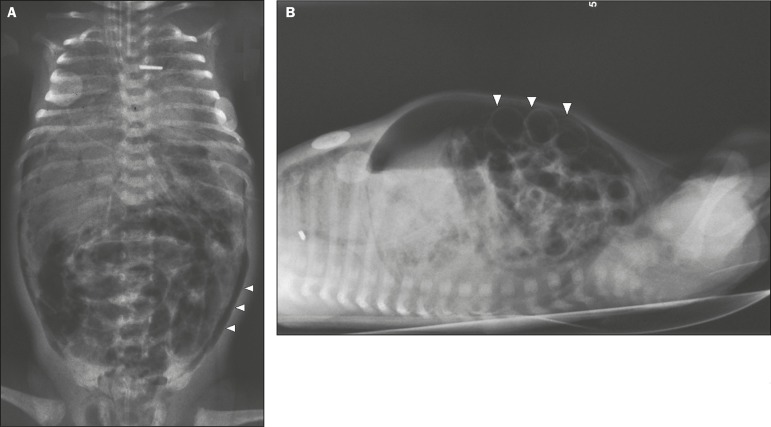
Premature newborn, at two days of life, presenting NEC. **A:** Conventional X-ray of the abdomen, in the supine position with vertical rays, showing distension of intestinal loops, intestinal pneumatosis, air in the portal system, and pneumoperitoneum (arrowheads). **B:** Conventional X-ray, in the supine position with horizontal rays, showing extensive pneumoperitoneum, visible between the anterior abdominal wall and the intestinal loops (arrowheads). Rigler’s sign (the walls of the intestinal loops visible) present. The newborn evolved to death.

**Figure 2:**
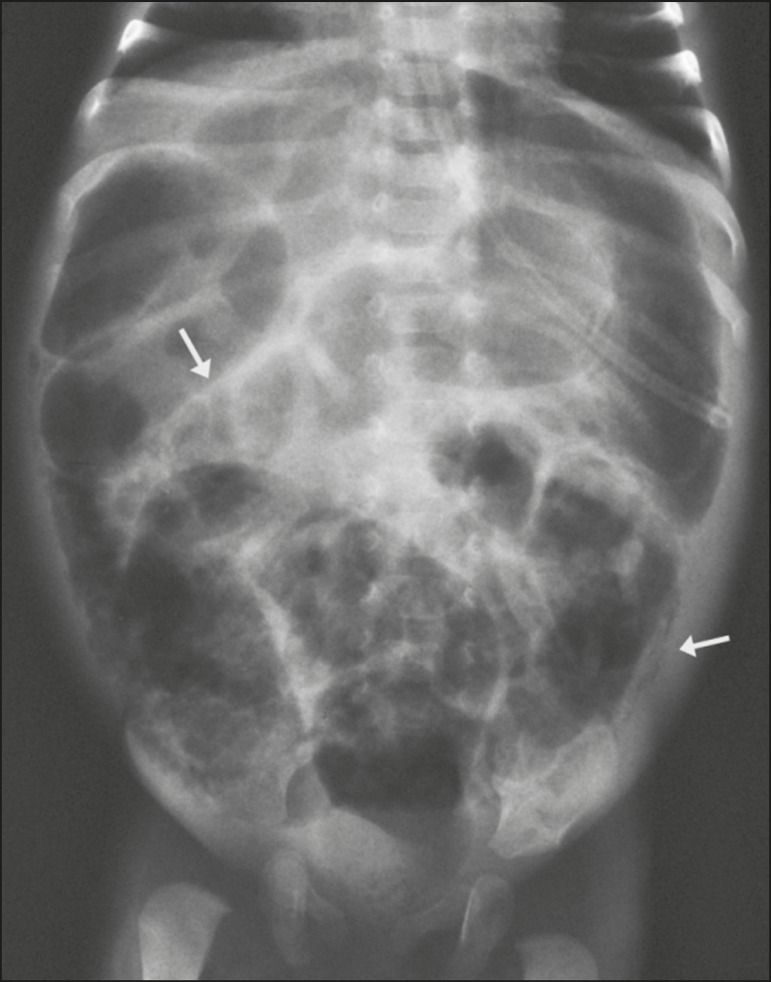
X-ray of a preterm newborn, in the supine position with vertical rays, indicating generalized distension of intestinal loops and pneumatosis (arrows) in segments of the large and small bowel.

**Figure 3:**
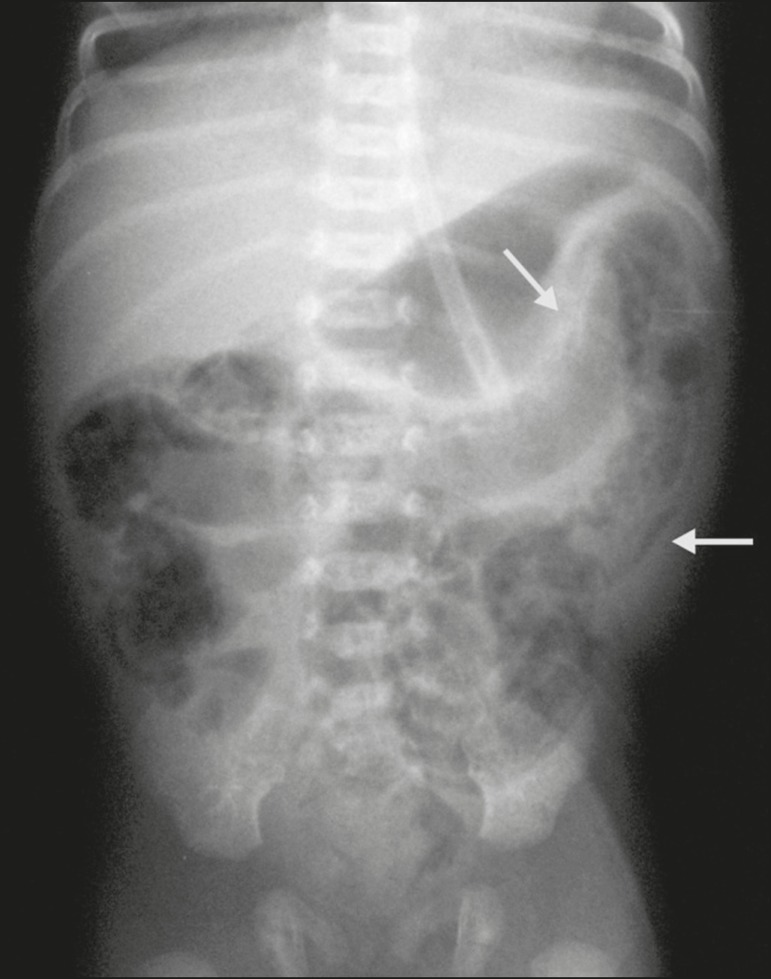
Conventional X-ray of the abdomen, in the supine position with vertical rays, showing pneumatosis in the large bowel (arrows). The newborn evolved with formation of stenotic lesions in the colon, requiring surgical correction at 38 days of life.

**Figure 4:**
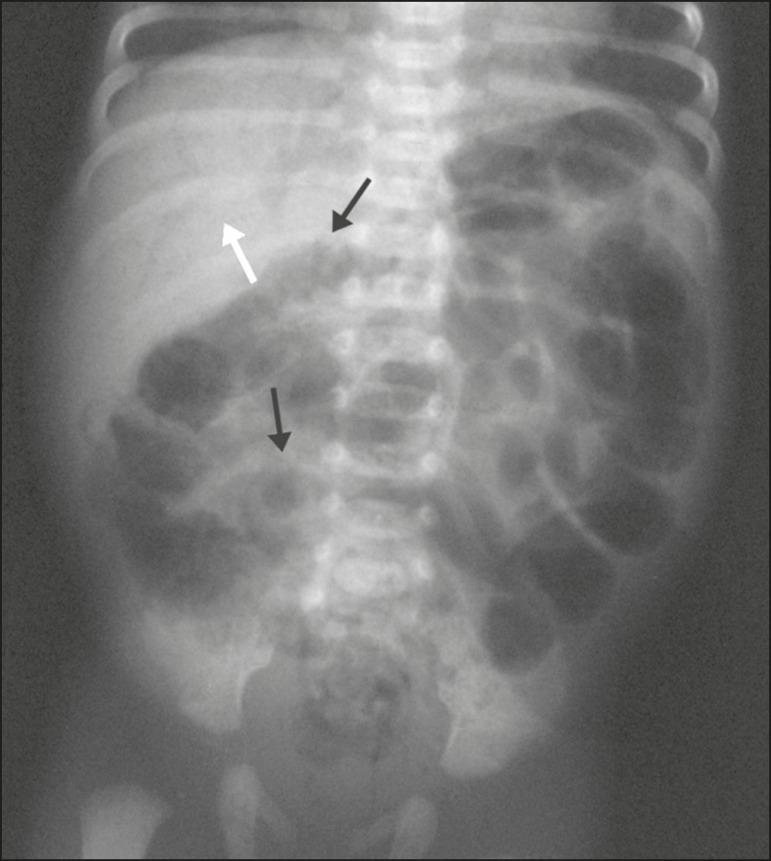
Conventional X-ray of the abdomen, in the supine position with vertical rays, of a preterm newborn showing intestinal pneumatosis (black arrows) and air in the portal system (white arrow). The newborn evolved to death at 31 days of age.

The independent radiological variables (Figures 1 to 4) were as follows: (a) the pattern of distribution of the intestinal pneumatosis, central and peripheral patterns likely corresponding to the small and large bowel, respectively; (b) the extent of the intestinal pneumatosis, classified as localized (restricted to one affected abdominal quadrant), moderate (two or three quadrants affected), or extensive (all four quadrants affected); (c) the morphology of the intestinal pneumatosis, the radiolucency being characterized as linear or bullous; (d) the presence of air in the portal system, defined as linear radiolucent branches that extend from the region of the main portal vein to the periphery of both hepatic lobes^(^^4^^)^; and (e) pneumoperitoneum.

The independent clinical variables studied were as follows: gestational age, established by the time of amenorrhea, by ultrasound, or by the Ballard score^(^^11^^)^; birth weight; gender; type of delivery; 5-min Apgar score; appropriateness of birth weight for gestational age ^(^^12^^)^; twinning; and chronological age when pneumatosis was identified. Additional variables were analyzed: the total duration of mechanical ventilation until the end of hospitalization, measured in days from the time of admission to the date of discharge; and duration of mechanical ventilation until the date on which pneumatosis was identified. The dependent clinical variable was death.

### X-ray examinations

Abdominal X-rays were obtained with a portable X-ray system (VMX plus; General Electric, Milwaukee, WI, USA), with the newborn in the supine position, in anteroposterior and profile views (vertical and horizontal rays, respectively).

### Data collection

Data collection was performed by the researcher responsible for the medical records review. The X-rays were evaluated by an experienced radiologist specializing in neonatal radiology. All of the X-rays available for each case were analyzed, with a minimum of two films per case. The worst finding was always given the most weight, and if there were two or more simultaneous findings, each was included in the statistical analysis independently. The combination of two simultaneous radiographic findings was not evaluated as a poor prognostic factor.

### Statistical analysis

Chi-square and Fisher’s exact tests were used in order to establish associations between categorical variables. The Mann-Whitney test was used in order to compare continuous variables. To analyze the factors associated with mortality, we used logistic regression analysis in the univariate and multivariate models, with a stepwise criterion of variable selection.

The significance level adopted for the statistical tests was 5%. The program used for statistical analysis was the Statistical Analysis System for Windows, version 9.4 (SAS Institute Inc., Cary, NC, USA).

## RESULTS

Of the 66 newborns analyzed, 56 (84.8%) were preterm, 57 (86.4%) had low birth weight, and 36 (54.5%) had very low birth weight. In 51 cases (78.5%), pneumatosis was restricted to the large bowel (peripheral pattern) and in 14 (21.5%) it was present in the large and small bowel. During the evolution, 7 newborns (10.6%) had air in the portal system, 15 (22.7%) had intestinal perforation, and 12 (18.2%) died. Of the 12 that died, 7 (58.3%) had perforation, 1 (8.3%) was a full-term newborn, and 9 (75%) had very low birth weight.

The bivariate analysis of the categorical variables between the newborns who survived and those who did not revealed a significant association between the occurrence of death and the simultaneous distribution of pneumatosis in the large and small bowel (central and peripheral patterns), pneumoperitoneum, and air in the portal system (Table 1). The newborns who died had a lower gestational age and a longer duration of mechanical ventilation, up to the identification of pneumatosis and throughout the hospital stay.

**Table 1:** Results of bivariate analyses for the outcome death.

Variable	Death (n = 12)	Survival (n = 54)	*P*-value
Birth weight (g), mean ± SD	1398.6 ± 699.0	1728.8 ± 769.9	0.065
Median	1175.0	1505.0	
Gestational age (weeks), mean ± SD	**30.9 ± 3.6**	32.8 ± 3.2	**0.043** *
Median	**30.0**	32.0	
5-min Apgar score, mean ± SD	8.3 ± 1.5	8.7 ± 1.4	0.36
Median	9.0	9.0	
Mechanical ventilation until discharge/death (days), mean ± SD	**19.0 ± 20.8**	4.3 ± 5.8	**< 0.0001** *
Median	**12.5**	1.5	
Mechanical ventilation until pneumatosis (days), mean ± SD	**10.0 ± 12.4**	1.8 ± 4.2	**0.0007** *
Median	**5.0**	0.0	
Chronological age at pneumatosis (days), mean ± SD	22.8 ± 16.2	17.2 ± 15.2	0.16
Median	18.5	11.5	
Male gender, n (%)	7 (58.3%)	26 (48.1%)	0.52
Cesarean delivery, n (%)	8 (66.7%)	39 (72.2%)	0.73
Small for gestational age, n (%)	5 (41.7%)	23 (42.6%)	0.95
Twin pregnancy, n (%)	1 (8.3%)	10 (18.5%)	0.67
Location of pneumatosis, n (%)			
Large bowel	5 (45.5%)	46 (85.2%)	**0.0089** *
Large and small bowel	**6 (54.5%)**	8 (14.8%)	
Extent of pneumatosis, n (%)			
Diffuse	4 (36.4%)	22 (40.7%)	0.42
Moderate	7 (63.6%)	24 (44.4%)	
Focal	0 (0.0%)	8 (14.8%)	
Morphology of pneumatosis, n (%)			
Bullous	3 (27.3%)	26 (49.1%)	0.32
Linear and bullous	8 (72.7%)	27 (50.9%)	
Air in the portal system, n (%)	**5 (45.5%)**	2 (3.7%)	**0.0010** *
Perforation of intestinal loops, n (%)	**7 (46.7%)**	8 (53.3%)	**0.0034** *

n, simple frequency; SD, standard deviation;

* Statistically significant.

Univariate logistic regression analysis identified the following variables as risk factors for death: time on mechanical ventilation until the identification of pneumatosis (odds ratio [OR] = 1.14; 95% confidence interval [95% CI] = 1.04-1.26), perforation of intestinal loops (OR = 8.05; 95% CI = 2.04-31.72), simultaneous localization of pneumatosis in small and large bowel (OR = 6.90; 95% CI = 1.69-28.10) and presence of air in the portal system (OR = 21.67; 95% CI = 3.43-137.08).

The results of the multivariate analysis are presented in Table 2. According to this analysis, the risk of death was highest among the patients who presented air in the portal system (69.7 times greater), perforation of intestinal loops (23.2 times greater), or pneumatosis with simultaneous central and peripheral distribution (12.4 times greater).

**Table 2:** Multivariate logistic regression analysis for mortality (n = 12).

Variable	Categories	*P*-value	OR	95% IC - OR
Air in the portal system	Yes vs. No (Ref.)	0.003	69.7	4.3-not calculated
Perforation of intestinal loops	Yes vs. No (Ref.)	0.009	23.2	2.2-246.7
Location of intestinal pneumatosis	Large and small bowel vs. large bowel only (Ref.)	0.035	12.4	1.2-127.4

OR, odds ratio; 95% CI - OR, 95% confidence interval - OR; Ref., reference level,

## DISCUSSION

In the present study, the best model to identify the mortality risk associated with NEC in newborns with a mean gestational age of 32.7 weeks was found to comprise the following radiological signs: air in the portal system; pneumoperitoneum; and central and peripheral distribution of pneumatosis.

In the bivariate analysis, the clinical variables gestational age and duration of mechanical ventilation (until the identification of intestinal pneumatosis and until discharge/death) differed between the newborns who survived and those who did not. In the univariate regression, the duration of ventilation until diagnosis was the only clinical factor indicative of greater risk, probably because it is a marker of greater clinical severity. Multivariate analysis, which is a powerful tool for suppressing confounding effects, discarded this clinical variable as a significant predictor of death. This finding differs from those of other authors^(^^2^^,^^13^^)^, which have shown that mortality is higher in newborns with lower gestational age, as well as in those with lower birth weight and those who are on mechanical ventilation on the day of NEC diagnosis. That discrepancy could be explained by differences in sample sizes and study designs. The study conducted by Clark et al.^(^^13^^)^, for example, analyzed only clinical variables, whereas the present study included radiological variables in the regression analysis.

There are controversies in the literature regarding the role that air in the portal system plays in the prognosis of NEC. Many authors have stated that air in the portal system and pneumoperitoneum are indicators of surgical necessity^(^^6^^,^^7^^,^^14^^)^. Surgical intervention, in turn, has been associated with higher mortality^(^^2^^)^. Some authors have found no association between air in the portal system and higher mortality^(^^9^^)^, whereas others have associated this finding with greater NEC severity^(^^8^^,^^15^^)^. The results of the present study are in accordance with the second observation, showing that air in the portal system was the variable associated with the highest risk of death.

Perforation of the intestinal loops is a well-studied variable established in the literature as an important risk factor for death^(^^16^^,^^17^^)^, acting as a marker of severity and of an immediate need for surgical intervention^(^^6^^,^^17^^)^. The relevance of this variable to the outcome death was corroborated by the present study.

Finally, our study revealed a significant association between the simultaneous distribution of pneumatosis in the large and small bowel (central and peripheral patterns) and death. Ultimately, broader distribution of pneumatosis indicates a greater extent of NEC involvement, corresponding to the greater extent of the inflammatory process secondary to the disease and resulting in a worse prognosis. It should be noted here that the variable of pneumatosis extension, evaluated by the abdominal quadrant criterion described in the literature^(^^8^^)^, and apparently easier to evaluate, was not significant in any of the statistical analyses. The large bowel is generally more difficult to identify in newborns, due to underdevelopment of the haustra^(^^18^^)^. We used the criterion of the site of the intestinal loops to recognize the two intestinal segments and, in this study; this criterion was more sensitive than was the criterion of the number of quadrants involved. This may be a consequence of transient pneumatosis, which can disappear rapidly, and of the extent of the pneumatosis, which can vary greatly over the course of the disease^(^^4^^)^.

In earlier studies of NEC, which were based on less sophisticated statistical analyses (i.e., simple frequency analyses), the radiological findings were often associated with the need for surgery and with mortality. We found few studies in which the radiological variables were analyzed by regression. This aspect seems to be a differential of our study. In contrast, Sharma et al.^(^^9^^)^, using the same method of statistical analysis, found that air in the portal system did not increase mortality, a result that was markedly different from that obtained in the present study. However, those authors used a sample in which very-low-birth-weight neonates predominated, air in the portal system and pneumatosis both being more common among the higher-weight infants in the same study. The same was reported in another study^(^^15^^)^. Therefore, air in the portal system, pneumatosis, and pneumoperitoneum are less common among lower birth weight newborns, consequently having lower sensitivity for the diagnosis and follow-up of NEC in this patient population.

According to the model obtained by the multivariate analysis, the newborns that were the most susceptible to death were those that presented air in the portal system, perforation of intestinal loops, and intestinal pneumatosis with simultaneous central and peripheral distribution. These findings explain the mortality rate, since they allow the inference that large sections of the intestines were affected, determining an unfavorable outcome. This association between death and extensive pneumatosis (isolated or with air in the portal system), with extensive intestinal necrosis, has previously been suggested^(^^8^^)^.

It is necessary to point out that the present study has limitations. Because the incidence of NEC at our facility (3.1%) is lower than that reported by other authors^(^^1^^-^^3^^)^, we evaluated only 66 cases over a 12-year period. This limited sample size had repercussions on the amplitude of the confidence intervals in the model generated by multivariate regression. Therefore, the amplitude of the confidence interval limited the precision of the risk assessment, but did not detract from the importance of the variable as a predictor of death, because the 95% CI was narrow in the univariate analysis, confirming the importance of each factor alone to the increase in mortality.

## CONCLUSION

Although long periods of mechanical ventilation prior to the appearance of intestinal pneumatosis represented a clinical risk factor for death, extensive intestinal pneumatosis, pneumoperitoneum, and air in the portal system made up the best set of factors associated with that outcome. Such an association corroborates the importance of conventional X-ray in the diagnosis and follow-up of NEC in newborns.
